# Strengthening the primary health care response to COVID-19: an operational tool for policymakers

**DOI:** 10.1017/S1463423621000360

**Published:** 2021-12-16

**Authors:** Anne S. Johansen, Amanda Shriwise, Daniel Lopez-Acuna, Pia Vracko

**Affiliations:** 1 Health Systems Strengthening, WHO Regional Office for Europe, Copenhagen, Denmark; 2 SOCIUM–Research Center on Inequality and Social Policy, University of Bremen, Bremen, Germany; 3 Department of Sociology, University of Kansas, Lawrence, United States; 4 Andalusian School of Public Health, Granada, Spain; 5 Department for Health Systems, National Institute of Public Health, Ljubljana, Slovenia

**Keywords:** COVID-19 response, governance, health service delivery, health system strengthening, primary health care, preparedness assessment

## Abstract

**Aims:**

The aim of this paper is to introduce an operational checklist to serve as a tool for policymakers in the WHO European Region to strengthen primary health care (PHC) services and address the COVID-19 pandemic more effectively and to present the results from piloting the tool in Armenia.

**Backgrounds:**

PHC has the potential to play a fundamental role in countries’ responses to COVID-19. However, this potential remains unrealized in many countries. To assist countries, the WHO Regional Office for Europe developed a guidance document – *Strengthening the Health Systems Response to COVID-19: Adapting Primary Health Care Services to more Effectively Address COVID-19* – that identifies strategic actions countries can take to strengthen their PHC response to the pandemic. Based on this guidance document, an operational checklist was developed to serve as a tool for policymakers to operationalize the recommended actions.

**Methods:**

The operational checklist was developed by transforming key points in the guidance document into questions in order to identify potentially modifiable factors to strengthen PHC in response to COVID-19. The operational checklist was then piloted in Armenia in June 2020 as part of a WHO mission to provide technical advice on strengthening Armenia’s PHC response to COVID-19. Two WHO experts performed semi-structured, face-to-face interviews with nine key informants (both facility managers and clinical staff) in three PHC facilities (two in a rural and one in an urban area). The data collected were analyzed to identify underlying challenges limiting PHC providers’ ability to effectively and efficiently respond to COVID-19 and maintain essential health services.

**Findings:**

The paper finds that making adjustments only to health services delivery will be insufficient to address most of the challenges identified by PHC providers in the context of COVID-19 in Armenia. In particular, strategic responses to the pandemic were missed, due, in part, to the absence of COVID-19 management teams at the facility level. Furthermore, the absence of PHC experts in Armenia’s national pandemic response team meant that health system issues identified at the facility level could not easily be communicated to or addressed by policymakers. The checklist therefore helps policymakers identify critical challenges – at both the facility and health system level – that need to be addressed to strengthen the PHC response to the COVID-19 pandemic.

## Introduction

Primary health care (PHC)^1^ has a fundamental role to play in countries response to the COVID-19 pandemic. It has the potential to care for the vast majority of COVID-19 patients close to their homes by informing patients about the disease, facilitating early diagnosis through testing, tracing of contacts, providing care to patients with mild or moderate cases of COVID-19, and referring patients for further care in the health system (WHO Regional Office for Europe, 2020a). It also plays an essential role in coordinating health services and ensuring continuity of care for patients with chronic diseases as well as maintaining other essential health services, which can help limit hospital stays and reduce the risk of hospitals exceeding their capacity. In this way, PHC can help rationalize the response to the pandemic and make the best use of scarce resources.

Despite its potential, patients in many countries bypassed PHC in the early stages of the pandemic, instead going directly to hospital. In some cases, this was the result of pandemic preparedness plans not having considered the potential role of PHC; in other cases, this occurred because the PHC system was able neither to serve as the population’s first point of contact with the health system nor to provide the PHC services that would enable it to function as the cornerstone of the health system (WHO and UNICEF, 2018).

To assist countries in strengthening their health system responses to COVID-19, the WHO Regional Office for Europe^2^ developed a series of technical guidance documents addressing specific health system issues (World Health Organization Regional Office for Europe, 2020c). One of these guidance documents, *Strengthening the Health Systems Response to COVID-19: adapting primary health care services to more effectively address COVID-19* (hereafter, ‘the WHO PHC COVID-19 Guidance’), published in June 2020, focuses on steps countries can take to strengthen the response of PHC to more effectively address the challenges created by the COVID-19 pandemic (World Health Organization Regional Office for Europe, 2020a). To support countries in the WHO European Region in operationalizing the recommended actions in the WHO PHC COVID-19 Guidance, a checklist was developed based on this guidance. This checklist was then piloted in Armenia during a WHO mission in June 2020.

The paper finds that making adjustments only to health services delivery will be insufficient to address most of the challenges identified by PHC providers in the context of COVID-19 in Armenia. These findings were subsequently mirrored in other information obtained by WHO in its work to assist countries in strengthening their response to COVID-19, indicating that the checklist, when used in conjunction with the WHO PHC COVID-19 Guidance, can serve as a useful tool to enable policymakers to identify and fill gaps in current PHC service delivery. As such, the checklist makes a useful contribution to the arsenal of tools available to countries in their fight against the pandemic.

By way of outline, the paper provides background information on the development of the WHO PHC COVID-19 Guidance. The methods section outlines the steps taken to develop the operational checklist and to pilot its use. Then, in the findings section, the paper provides an overview of the main sections of the checklist and the results from the pilot in Armenia. The discussion section analyses the results from the pilot in Armenia and the lessons learned. The paper then concludes by reflecting on how the operational checklist could be further developed and used in other countries.

### Background: the WHO PHC-COVID-19 Guidance

The WHO PHC COVID-19 Guidance was prepared on the basis of a systematic review of the best available evidence and emergent country practices in response to the COVID-19 outbreak in the WHO European Region (World Health Organization Regional Office for Europe, 2020a).^3^ The guidance was developed specifically to aid policymakers in countries in addressing managerial and organizational aspects of PHC in response to the pandemic; it is not intended to provide clinical advice about the treatment of COVID-19 patients.^4^ The guidance document identifies three strategic directions for strengthening PHC services in countries to better address COVID-19: i) integrating PHC more prominently into the overall public health response; ii) adapting the roles and responsibilities of PHC; and iii) maintaining the delivery of essential, non-COVID-19 PHC services during the pandemic.

In addition, the WHO PHC COVID-19 Guidance also includes a section on system-level recommendations to ensure the presence of the enabling conditions needed for effective implementation of the recommended actions. This section covers the six essential health systems’ building blocks^5^ that need to be considered when adopting a PHC approach in order to meet the new requirements for managing COVID-19 (World Health Organization, 2007). Evidence suggests that all health system building blocks must be considered. Narrowing the focus to, for example, health service delivery while ignoring the need for adequate resources such as human, financial, and information systems, risks limiting the ability to identify performance issues in the organization, management, and delivery of PHC services.

## Methods

The WHO multidisciplinary expert team that developed the WHO PHC COVID-19 Guidance also prepared the checklist^6^, building upon its structure (please see Supplementary Material). However, in contrast to the WHO PHC COVID-19 Guidance, the checklist first addresses system-level issues in recognition of the crucial role they play in ensuring effective implementation of actions under each strategic direction. This placement is also meant to signal the importance of applying the checklist to all aspects of the dimensions of the health system not only to one or more of its component parts.

The checklist consists of questions that correspond to the specific recommended actions under the three strategic directions and the system-level section of the WHO PHC COVID-19 Guidance, in order to identify areas where actions could be taken to strengthen the PHC response to COVID-19. The operation checklist was developed by transforming key points made in the main text of the WHO PHC COVID-19 Guidance, as well as those summarized in its tables, into questions that could be answered categorically as ‘Yes’, ‘No’, or ‘Partially’, with an additional column for comment.^7^

Regarding case selection, the checklist was piloted in Armenia in June 2020 after the government made an urgent request to the WHO Regional Office for Europe for guidance on how to strengthen their PHC response to COVID-19. While this case selection was pragmatically driven, its results provide useful insights into the value of the checklist and are of particular relevance to many other countries.

Due to travel restrictions related to COVID-19, only one expert from the WHO European Health Emergency Team was able to travel to the pilot country to work with the local WHO expert from the WHO Country Office in Armenia, while the rest of the mission members participated virtually. The regional and local WHO experts carried out semi-structured, face-to-face interviews with nine key informants (both facility managers and clinical staff) in three PHC facilities (two in a rural and one in an urban area) visited by the WHO mission. All informants agreed to participate in the pilot. Data were collected by manual note taking. After each meeting, the preliminary data were shared with the other mission members, including other experts from the WHO Country Office and members working at a distance, to clarify emergent questions and/or to obtain additional information. Information was also obtained from key informants in the Ministry of Health and the National Institute of Public Health. Lessons learned from the discussions during the virtual meetings also helped to revise and further develop the checklist. The data collected were analyzed after the mission to develop recommendations.

## Findings

The findings are presented in two parts. The first details the structure of the operational checklist based on the WHO PHC COVID-19 Guidance, and the second outlines the results of its use in Armenia.

### Operational checklist to strengthen PHC response to COVID-19 in countries

The PHC operational checklist consists of four sections, each focused on a key objective for strengthening PHC during the COVID-19 response in countries: i) build system-level implementation capacity; ii) integrate PHC more prominently into the overall public health response to COVID-19; iii) adapt the roles and responsibilities of PHC to better respond to COVID-19; and iv) maintain the delivery of essential (non-COVID-19) PHC services during the pandemic. A copy of the PHC operational checklist can be found in the supplementary material.

The first section, ‘Build system-level implementation capacity’, assesses practices in five key domains: i) bridge the governance mechanism of the emergency response and the governance mechanism for PHC; ii) adopt policies to adequately resource PHC services; iii) take steps to ensure adequate levels of properly trained human resources during peak periods of the epidemic; iv) effectively protect the PHC workforce; and v) strengthen logistic capabilities to ensure the supply chain. Together, the questions posed in this section of the checklist explore the PHC system-level strengths and weaknesses in order to identify possible gaps. Examples of system-level gaps may include the lack of a PHC governance mechanism and its relationship to public health services at both national and local levels; an inadequate supply of personal protective and medical equipment; and the absence of health workforce strategic planning.

The second section, ‘Integrate PHC more prominently into the overall public health response to COVID-19’, assesses practices in five key domains: i) inform patients and the community about COVID-19; ii) interrupt the chain of transmission of the virus; iii) enhance precision and reach of epidemiological surveillance; iv) identify and protect vulnerable and at-risk individuals and population groups; and v) ensure appropriate referrals for testing, home isolation, and hospital admission. Together, the questions posed in this section of the checklist explore the extent to which public health services are being delivered to individuals at the PHC level and to identify areas where further integration is needed. Examples of gaps in integration may include PHC providers focusing predominantly on treating COVID-19 without delivering essential services and PHC providers failing to communicate basic information on COVID-19 protective measures (e.g. coughing hygiene, use of hand disinfectants, wearing masks, and physical distancing), testing, isolation, and quarantine with cultural and linguistic sensitivity.

The third section, ‘Adapt the roles and responsibilities of PHC to better respond to COVID-19’, assesses practices in five key domains: i) establish COVID-19 testing sites outside of health care facilities; ii) separate care pathways for COVID-19 and other patients; iii) develop new service delivery modalities and innovate platforms and tools; iv) organize and supervise the care of patients staying at home or in alternative care settings; v) and strengthen the interface with the care for people in nursing homes and other closed settings such as refugee camps, prisons, and detention centres. Together, the questions posed in this section of the checklist explore the flexibility of PHC services to adapt to the new needs related to COVID-19. Challenges and problems from lack of adaptation may include PHC providers not accepting COVID-19 patients for fear of spreading the virus to health workers and non-COVID-19 patients; reduced access to PHC due to insufficient application of digital health tools; and disruption to follow up with patients at home or in other closed settings, which is particularly problematic for COVID-19 patients at high risk of developing severe COVID-19.

The fourth section, ‘Maintain the delivery of essential, non-COVID-19 PHC services during the pandemic’, assesses practices in three key domains: i) review and revise the scope of PCH services to be provided during the epidemic to maximize the ability to respond to the COVID-19 epidemic while preserving essential services; ii) develop new modalities of work and service delivery to facilitate business continuity of regular PHC; and iii) develop innovative tools and mechanisms to reduce the burden on PHC providers. The questions posed in this final section of the checklist explore the extent to which PHC services are fit to continue providing essential preventative and curative care to non-COVID-19 patients. If essential PHC service delivery is not fully maintained, this may be, in part, because individuals are reluctant to use PHC. Furthermore, essential PHC services may face greater, and even overwhelming, demand after prolonged lockdowns and/or high prevalence of COVID-19. Examples of problems that may arise when essential service delivery is not maintained may include inadequate follow-up with patients with chronic diseases and/or mental health problems; and delayed diagnosis, which may result in increased unmet health needs, disease progression, and potentially, worse health outcomes.

### Results from the use of the operational checklist in *Armenia*

The pilot of the operational checklist in Armenia revealed several important points about its usefulness as a tool for strengthening the PHC response to COVID-19 in countries. First, administrating the checklist by interview revealed contextual elements that are critical to understanding Armenia’s PHC response to COVID-19. In a proactive effort to manage the pandemic and minimize the risk of hospitals being overrun by COVID-19 patients, the Government of Armenia issued a Ministerial Decree in May 2020 that changed the management of laboratory-confirmed COVID-19 patients with mild or no symptoms from isolation in hospital to isolation at home. The Decree also established the functions and accountability of PHC providers in the COVID-19 response and created standards for suspected and confirmed COVID-19 cases as well as their contacts. The changes dictated by the Decree led to a growing number of COVID-19 patients being managed at the PHC level, which brought about a rapid escalation in the workload of the PHC health workforce, particularly in large urban areas, such as Yerevan, where the community spread was greatest. As a result, PHC providers began voicing concern about excessive workloads and being near or at full capacity. Local reports of declining rates of childhood immunizations also began to appear, in line with early evidence from other countries indicated that maintaining essential services was becoming increasingly difficult in context of a high number of new COVID-19 patients.^8^ While it may be possible to arrive at a similar level of understanding about a particular context or country case through the use of the comment box in the checklist if administrated as a survey, administrating the checklist in the style of a semi-structured interview proved to be a useful and efficient way to gain critical information about the context in which the PHC response to COVID-19 is taking place.

Second, the responses to the checklist questions revealed that the Government of Armenia has made a significant commitment to position PHC as a meaningful first point of contact for its population during the COVID-19 pandemic and that it has taken a pragmatic and flexible approach to repurposing its health services to prevent and manage the pandemic. The responses also indicated that despite numerous efforts to ensure the continuity of essential health services while also carrying out their COVID-19 responsibilities, PHC centers in the areas hardest hit by the pandemic were overstretched and struggling to keep up with the demand for their services. In particular, strategic responses to the pandemic were missed, due, in part, to the absence of COVID-19 management teams at the facility level. Furthermore, the absence of PHC experts in Armenia’s national pandemic response team meant that health system issues identified at the facility level could not easily be communicated to or addressed by policymakers.

Together, administration of the checklist identified eight health system challenges limiting PHC providers’ ability to effectively and efficiently respond to COVID-19 and maintain essential health services:

1. An inadequate surge capacity to ensure a sufficient number of staff in PHC centers most affected by the pandemic;
2. An ineffective/inefficient use of the PHC health workforce;
3. The continued delivery of non-essential, non-COVID-19 services, which reduced the time available to manage the increased workload due to COVID-19;
4. A suboptimal organization of COVID-19 testing services at the PHC level;
5. Suboptimal service delivery platforms and work modalities;
6. Excessive reporting requirements related to COVID-19 patients that were of limited use to PHC providers;
7. The absence of a facility-based team to regularly monitor the situation and take action to address emerging problems; and
8. The absence of a mechanism to communicate problems that require system-level intervention to address them.

By specifying these challenges, the checklist enabled the development of recommendations to address them (Box 1). In this way, the checklist proved useful in translating WHO PHC COVID-19 Guidance into context-specific recommendations for improving Armenia’s PHC response to COVID-19, thereby filling the main aim of the operational checklist. However, the nature of the application of the operational checklist during a WHO mission does not allow for an evaluation of the extent to which the PHC response to COVID-19 has improved as a result of using the operational checklist. Further research will be required to assess the impact of such evaluations tools in the COVID-19 response and how best to use them, which is critical for informing the PHC response to future health emergencies.

Box 1. Eight steps towards a more effective PHC response to COVID-19 in Armenia – A summary of recommendations

1. Ensure an adequate number of staff in the PHC centers most affected by the pandemic to meet the increased demand.
  - Extend the workday of the existing PHC workforce to increase PHC capacity to respond to the COVID-19 pandemic.
  - Address the reluctance of PHC staff to be reallocated from localities less affected by COVID-19 to those more affected. Reluctance to be reallocated may stem from but is not limited to: fear; lack of authority; and lack of understanding of the importance of the reallocation.
  - Use WHO’s surge capacity calculator (World Health Organization, 2020b) to predict the additional workforce capacity that will be needed.
  - Strengthen collaboration between the national pandemic response team and the PHC authorities to address PHC system issues (e.g. workforce capacity, monitoring and responding to new developments).
2. Use the PHC health workforce more effectively and efficiently.
  - Use innovative means to relieve the burden on PHC workers. This could be done by, for example, mobilizing community health workers, collaborating with NGOs and patient associations, retraining of existing health care workers, and training non-medical personnel for functions that do not require medical training.
  - Increase the validity of prescriptions for patients with well-controlled NCDs, such as hypertension and diabetes.^9^
  - Shift all possible tasks to the lowest-skilled member of the PHC team to free up the time of physicians and nurses to provide the services that no one else can.
  - Ensure that the care provided follows evidence-based guidelines that are up to date, both for COVID-19 and non-COVID-19 patients.
  - Delegate telephone follow-up with COVID-19 patients in home isolation to staff from PHC centers in localities less affected by the pandemic, freeing up staff in the local PHC centers to perform other, more critical tasks.
3. Eliminate non-essential, non-COVID-19 services.
  - Develop a governmental policy to limit access to non-essential PHC services.
  - Create a roadmap with defined trigger/thresholds for a phased reallocation of routine comprehensive service capacity toward the most essential services. This will enable PHC providers to maintain quality of care for both COVID-19 management and the delivery of essential services by reducing demand.
4. Optimize the organization of COVID-19 testing services at the PHC level.
  - Develop alternative testing sites that are not PHC facilities, such as stand-alone, drive-in testing facilities, mobile testing services that go to people’s homes to collect testing, and/or testing at the National Centre for Disease Control.
5. Develop novel service delivery platforms and new work modalities.
  - Introduce innovative new modalities of work and service delivery that are more cost effective and accessible. This may include, but is not limited to: introducing telemedicine solutions and outreach mechanisms, such as clinical e-consultations conducted via video chat or text message; staffed helplines; e-pharmacies that allow remote/electronic prescriptions; and mobile clinics with remote connections to health facilities for timely access to patient data such as medication lists and diagnostic test results.
  - Leverage digital health technologies to increase medication adherence and to empower individuals to take more proactive measures to manage their own health (World Health Organization, 2020, 2019).
6. Reduce the burden on PHC providers by reducing reporting requirements.
  - Review and revise the reporting process to minimize the burden on PHC physicians while ensuring that the essential information about COVID-19 patients is maintained and used.
7. Establish a facility-based team to regularly monitor the situation and empower it to take action to address emerging problems.
  - Establish a COVID-19 management team to monitor the situation on a daily basis.
  - Delegate increased authority to PHC directors to take action and address challenges in the best way possible.
  - Establish a direct line of contact between PHC directors and the national PHC authority to communicate problems that require system-level intervention, such as an update of existing regulations, guidance documents, and protocols.
8. Establish a national committee to monitor the performance of the PHC response to COVID-19 and to address problems that require system-level intervention.
  - Establish a national committee with members from both public health and PHC authorities as well as other relevant stakeholders.
  - Create terms of reference for the committee that includes monitoring the performance of PHC providers, both in terms of managing COVID-19 patients and ensuring that essential health services are maintained and serving as the point of contact for PHC facilities that have encountered challenges requiring system-level intervention.

## Discussion: lessons learned from applying the operational checklist in Armenia

The pilot of the operational checklist in Armenia demonstrated its utility as a tool to identify both the strengths and weaknesses of Armenia’s efforts to place PHC at the center of the country’s response to the COVID-19 pandemic. In particular, it identified eight specific areas where there are opportunities for strengthening the role and effectiveness of PHC’s response to the pandemic. When used in conjunction with the WHO PHC COVID-19 Guidance, the operational checklist helped to develop context-specific recommendations for addressing the challenges faced by Armenia’s PHC system, and, potentially, to increase the effectiveness of its pandemic response by better utilizing scarce human resource while also making health services more person-centered.

The most important technical insight generated from piloting the operational checklist is that making adjustments only to health services delivery will be insufficient to address most of the challenges identified by PHC providers in Armenia. While some actions can be taken by facility managers and clinical professional, system-level interventions by governments will also be required if the main challenges identified are to be addressed. For example, ensuring an adequate number of staff in PHC centers to meet the increased demand created by COVID-19 in Armenia will require a number of actions that are beyond the capacity and jurisdiction of individual PHC providers. Furthermore, required actions, such as increasing the length of the workday, changing the salaries/bonuses that the health workforce staff are paid and/or modifying the roles and responsibilities of different categories of PHC staff (e.g. doctors, nurses, and other health workers) can only be taken by the central government.

Furthermore, the application of the checklist in Armenia highlights the need for a mechanism at the national level to monitor the impact of COVID-19 on the PHC system and to ensure that action is taken when health system constraints undermine the ability of PHC to manage its COVID-19 response. It also suggests a need for greater integration of experts in health systems and PHC practitioners into the national pandemic response team to coordinate the myriad initiatives required for effective management of the pandemic and to ensure that the necessary resources (both human and financial) are available and used efficiently and effectively.

Moreover, the results from the pilot also demonstrate that although the checklist was developed as a stand-alone tool, there is a need for more detailed instructions on how it can be used. While it would have been desirable to obtain input from stakeholders at both the facility level and within the central government, particularly because the findings revealed that addressing challenges at the facility level will require intervention at the national/system level, this was not possible given the time constraints of both the mission and the ongoing pressures for responding to pandemic. Regional or local governments should also be consulted in proportion to the role they play in managing the PHC system. Also, while the administration of the checklist as an interview provided critical information on the context in which Armenia’s PHC response was taking place, more systematic feedback from a range of stakeholders could be gained by administrating the checklist as a survey, and this should be further explored in additional pilot study’s to inform future use of the operational checklist.

## Conclusion: what can other countries learn from the pilot of the operational checklist in Armenia?

Based on the WHO PHC COVID-19 Guidance, a checklist was developed as an operational tool to assist countries in the WHO European Region in strengthening the response of their PHC systems to COVID-19. As such the tool is generic and thus relevant to all countries, and it is designed to facilitate the development of context-specific policy recommendations for actions to strengthen the PHC response to COVID-19.

The application of piloting the operational checklist in Armenia has shown that the tool can be used to identify both facility and system-level challenges that need to be addressed to strengthen a country’s PHC response to the COVID-19 pandemic. To increase the likelihood that the challenges identified through the application of the checklist are actually addressed, it is essential that the checklist is used in participatory manner and as part of a systematic effort to monitor the performance of the PHC system during the COVID-19 pandemic, and there must also be mechanisms to ensure that action is taken when needed. While it is beyond the scope of this paper to identify the most effective mechanism to ensure that action is taken, experience would suggest that there needs to be a working group or task force on PHC issues comprised of key stakeholders at the system, local, and facility levels. It will also be necessary to ensure that there is a formalized method to coordinate the activities of PHC with the national pandemic response. Establishing a PHC task force as a working group under the national pandemic response committee and/or including key PHC stakeholders in the national pandemic response team are a couple of ways to do this, but other options are also possible. The important thing is for countries to find governance mechanism(s) that are well suited to their context.

In countries where PHC has yet to evolve into the fully developed cornerstone of a sustainable health system, the operational checklist and the accompanying WHO PHC COVID-19 Guidance may serve as tools to strengthen the capacity of the government to improve the performance of their PHC system, not only during COVID-19 but throughout recovery and transition and even beyond. As such, the checklist serves as a potentially important contribution to the arsenal of tools available to countries in their fight against the pandemic.

## Footnotes

1 The term PHC is used according to the definition of the European Expert Panel on Effective Ways of Investing in Health and the Declaration of Astana (European Commission, 2014; World Health Organization and the United Nations Children’s Fund, 2018).

2 The WHO Regional Office for Europe provides technical support to countries throughout the WHO European Region, which includes 53 member states. For a list of these countries, please see: https://www.euro.who.int/en/countries.

3 The WHO PHC-COVID-19 Guidance targets PHC policymakers and addresses issues relevant for PHC providers; it does not address the role of community pharmacists, dentists, other health professionals or social service providers in the PHC COVID-19 response.

4 A number of other WHO guidance documents have been developed for this purpose. For example, please see WHO (2020c).

5 The six essential health system building blocks are i) services delivery; ii) health workforce; iii) information; iv) medical products, vaccinations, and technologies; v) financing; and vi) leadership and governance (World Health Organization, 2007).

6 The first, third, and last authors of this paper also co-authored the WHO PHC-COVID guidance and the checklist.

7 Beyond the WHO Regional Office for Europe’s Technical Guidance #5 alone, two substantive additions were made to the operational checklist, as relevant guidance documents continued to evolve and be updated. In section 3, subsection 3.1 (‘Establish COVID-19 testing sites outside of health care facilities’). In sub-section 1.4 (‘Effective protect the PHC workforce’), a question was added regarding the weekly screening and testing of health care workers.

8 It was in this context that the Government of Armenia sought guidance from the WHO Regional Office for Europe on how to optimize its PHC services to strengthen its response to COVID-19 and ensure effective management of asymptomatic and mild cases of COVID-19.

9 In some countries, such patients are issued 3-month prescriptions with four refills so that it is not necessary to see a PHC provider for a whole year.

## Supplementary material

For supplementary material accompanying this paper visit http://dx.doi.org/10.1017/S1463423621000360. click here to view supplementary material
