# Supplementary material for: Strengthening the primary health care response to COVID-19: an operational tool for policymakers
Source: Prim Health Care Res Dev. 2021 Dec 16;22:e81. doi: 10.1017/S1463423621000360 (PMC8695943; doi:10.1017/S1463423621000360)
Supplement: Supplementary file 1 [file S1463423621000360sup001.docx]

**Supplementary material: Operational checklist to strengthen PHC response to COVID-19 in countries**

| **1. Build system-level implementation capacity** | | | | |
| --- | --- | --- | --- | --- |
| **1.1 Bridge the governance mechanism of the emergency response and the governance mechanism for primary health care (PHC)** | **Yes** | **No** | **Partly** | **Comments** |
| - - 1. Is there an explicit governance mechanism for PHC services at the national and subnational levels? |  |  |  |  |
| - - 1. Is there an explicit bridge between the governance mechanism of the emergency response and the governance mechanism for PHC services at national and sub-national levels? |  |  |  |  |
| - - 1. Is there a mechanism to oversee the development and adaptation of policies, regulations, and standard operating procedures to enable PHC providers to respond more effectively to COVID-19 and retain functionality to provide essential health services? |  |  |  |  |
| - - 1. Are service delivery patterns – particularly for essential health services – being carefully monitored in order to coordinate reprioritization and the reorientation of referral pathways, working through relevant authorities as well as public and private service providers? |  |  |  |  |
| - - 1. Are resource needs being monitored and adequate financing been mobilized to support the intensified PHC response and needed health system inputs? |  |  |  |  |
| - - 1. Are the needs of vulnerable and marginalized populations, including migrants and refugees, being considered when developing national responses to the pandemic? |  |  |  |  |
| - - 1. Is the pandemic and the ability of the PHC system to effectively tackle it being monitored/analysed, ensuring that corrective actions are taken, when needed? |  |  |  |  |
| - - 1. Has the stewardship of PHC been strengthened so that all PHC providers engage in service provision and coordinate with public health infrastructure? |  |  |  |  |
| - - 1. Has a functional mapping of PHC facilities, including those in public and private systems, been conducted? |  |  |  |  |
| - - 1. Are multiple PHC practices (when applicable) being linked so single practitioner or small group practices are able to liaise with other practices in neighbourhoods to support each other and establish shared services (e.g., clinics for suspected cases)? |  |  |  |  |
| **1.2 Adopt policies to adequately resource PHC services** | **Yes** | **No** | **Partly** | **Comments** |
| - - 1. Is PHC financing for both COVID-19 and essential health services adequate and ensured (e.g., increasing existing budget allocations, reshaping PHC purchasing models)? |  |  |  |  |
| - - 1. Have all PHC services been made free of charge to prevent financial hardship? |  |  |  |  |
| - - 1. Is population empanelment, where all persons have an identified PHC provider, being expanded/strengthened to ensure universal access to PHC, particularly vulnerable and high-risk groups, including migrants, refugees and asylum seekers? |  |  |  |  |
| **1.3 Take steps to ensure adequate levels of properly trained human resources during peak periods of the epidemic** | **Yes** | **No** | **Partly** | **Comments** |
| - - 1. Are additional community resources (e.g., networks of trained community health workers) being mobilized? |  |  |  |  |
| - - 1. Is their collaboration between the government and nongovernmental organizations (NGOs) and patient associations? |  |  |  |  |
| - - 1. Are existing health care workers being retrained to ensure adequate care provision for more severely ill COVID-19 patients? |  |  |  |  |
| - - 1. Are health care workers who are not currently working in the system being brought in and trained for new tasks and safety to increase the pool of professional available to support PHC provision and the health system? |  |  |  |  |
| - - 1. Are nonmedical personnel being trained to carry out functions (e.g., temperature and blood pressure measurements) to alleviate the burden on other health care providers? |  |  |  |  |
| - - 1. Has a community health workforce been mobilised and properly trained to assist in this? |  |  |  |  |
| **1.4 Effectively protect the PHC workforce** | **Yes** | **No** | **Partly** | **Comments** |
| - - 1. Are all PHC health workers being trained on infection prevention and control measures, including application of standard and transmission-based precautions, rational use of personal protective equipment, etc.? |  |  |  |  |
| - - 1. Are health workers being screened and tested on a weekly basis, irrespective of symptoms to reduce contagion as well as the fear and anxiety both of working on the frontline? |  |  |  |  |
| - - 1. Are the families of PHC health workers being protected by offering alternative accommodation for health workers that are working directly with COVID-19 patients? |  |  |  |  |
| - - 1. Do all health care institutions that come into direct contact with patients, such as PHC facilities, have protocols of action that are regularly reviewed, and contingency plans for operating in acute crisis situations? |  |  |  |  |
| - - 1. Are staff members who have long-term medical problems (e.g., those on immune-suppressors, cancer treatment, diabetes, hypertension or asthma) being identified and moved to non-patient-facing roles? |  |  |  |  |
| - - 1. Is the mental health and well-being of the PHC workforce being protected (e.g., setting up a mental health hotline to support and advise health workers during the pandemic)? |  |  |  |  |
| **1.5 Strengthen logistic capabilities to ensure the supply chain** | **Yes** | **No** | **Partly** | **Comments** |
| - - 1. Are supplies of essential medicines and health technologies adequate and ensured? |  |  |  |  |
| - - 1. Are national guidelinesrecommending medicines for the management of COVID-19 infection beingupdated regularly? |  |  |  |  |
| - - 1. Is there a central mechanism to ensure awareness of problems with the supply of commonly used medicines and are suitable levels of buffer stocks available to ensure that supply of regular customers is maintained? |  |  |  |  |
| - - 1. Is there a mechanism to ensure continuity of supply for chronic medical conditions and are pharmacists aware of any special provisions to enable supply of essential medicines (e.g., asthma inhalers, contraceptives, antihypertensive medicines and medicines for heart and kidney disease)? |  |  |  |  |
| - - 1. Is an adequate supply of personal protective equipment (e.g., masks, goggles or face shields, gloves, gowns, hand hygiene supplies, and products for cleaning and disinfection of environmental and medical equipment) being ensured and made readily available? |  |  |  |  |
| - - 1. Is an adequate supply of diagnostic tests and kits for taking the samples being ensured? (This includes diagnostic equipment, reagents and trained personnel as well as the means to perform, process and quickly communicate the results) |  |  |  |  |
| **2. Integrate PHC more prominently into the overall public health response to COVID-19** | | | | |
| **2.1 Inform patients and the community about COVID-19** | **Yes** | **No** | **Partly** | **Comments** |
| - - 1. Are PHC policy makers and providers collaborating with public health officials to develop and implement culturally and linguistically sensitive population information regarding COVID-19 symptoms, how to limit transmission of virus, and where to receive additional information? |  |  |  |  |
| - - 1. Are PHC providers contributing to efforts to provide a clear first point of contact entry point into the health system to receive advice when presenting with COVID-19 symptoms or other related concerns? |  |  |  |  |
| - - 1. Are PHC policy makers and providers collaborating with public health officials in the establishment and, possibly, staffing of COVID-19 information hotlines? |  |  |  |  |
| **2.2 Interrupt the chain of transmission of the virus** | **Yes** | **No** | **Partly** | **Comments** |
| - - 1. Are PHC providers educating health care workers, social service providers, employees in nursing homes and other frontline workers about infection prevention and control measures to protect themselves and their clients against virus transmission? |  |  |  |  |
| - - 1. Are PHC providers communicating culturally and linguistically appropriate public health information on infection prevention and control measures, such as hygienic practices, proper use and handling of protective masks and physical distancing to patients and the community to increase health literacy and reduce the transmission of the virus? |  |  |  |  |
| **2.3 Enhance the precision and reach of epidemiological surveillance** | **Yes** | **No** | **Partly** | **Comments** |
| - - 1. Are PHC providers supporting epidemiologic surveillance by promptly reporting confirmed and suspected cases to public health/epidemiologic services? |  |  |  |  |
| - - 1. Are PHC providers supporting epidemiologic surveillance by assisting with contact tracing of suspected and confirmed cases? |  |  |  |  |
| - - 1. Are PHC providers supporting epidemiologic surveillance by reaching out to patients and people in home isolation on a regular basis to help them cope with the required isolation? |  |  |  |  |
| **2.4 Identify and protect vulnerable and at-risk individuals and population groups** | **Yes** | **No** | **Partly** | **Comments** |
| - - 1. Are PHC providers reaching out to older and institutionalized peopleto ensure early detection and appropriate measures to prevent transmission? |  |  |  |  |
| - - 1. Are PHC providers reaching out to other at-risk people, such as those suffering from a noncommunicable disease (NCD) or any other co-morbidity, to ensure early detection and appropriate measures to prevent transmission? |  |  |  |  |
| - - 1. Are PHC providers reaching out to other vulnerable persons and groups, such as those undergoing financial hardship, large families living amid material deprivation,and those with low literacy levels to ensure public health messages are being heard and understood? |  |  |  |  |
| - - 1. Are PHC policy makers and providers working with public health officials and other relevant authorities to develop tailored public health measures for migrants, refugees and others living in close proximity and who, as a result, are at greater risk for transmitting the virus and are unable to self-isolate? (Such measures might include establishing new, less crowded living arrangements and, in the case of infected persons, referral to alternative care sites, such as converted schools or convention centres) |  |  |  |  |
| - - 1. Are PHC providers working with public health officials and community services to ensure access to essential medicines and health technology for those in isolation or quarantine, particularly individuals living alone or with limited mobility? |  |  |  |  |
| - - 1. Are PHC providers working with public health and community services to ensure the provision of food for those in isolation or quarantine, particularly individuals living alone or with limited mobility? |  |  |  |  |
| **2.5 Ensure appropriate referrals for testing, home isolation and hospital admission** | **Yes** | **No** | **Partly** | **Comments** |
| - - 1. Are PHC policy makers and providers working jointly with public health officials on the development and implementation of protocols for isolation and quarantine, tracing of contacts, referral of individuals with the highest probability of poor outcomes to hospital? |  |  |  |  |
| **3. Adapt the roles and responsibilities of PHC to better respond to COVID-19** | | | | |
| **3.1 Establish COVID-19 testing sites outside of health care facilities** | **Yes** | **No** | **Partly** | **Comments** |
| - - 1. Have testing sites been established in the most appropriate way(s) (e.g., drive through testing sites or designated tent areas)? |  |  |  |  |
| - - 1. Have mobile teams been established to enable testing at home for people with limited mobility? |  |  |  |  |
| - - 1. When appropriate, are PHC providers collecting specimens for testing? |  |  |  |  |
| - - 1. Is safe transit available to PHC providers or testing sites? |  |  |  |  |
| **3.2 Separate care pathways for COVID-19 and other patients** | **Yes** | **No** | **Partly** | **Comments** |
| - - 1. Are patient pathways being separated and clarified to facilitate the most efficient use of health care resources, optimize the existing network of PHC providers and establish new health care settings for confirmed or suspected patients, if needed? |  |  |  |  |
| - - 1. Are all patients being screened on arrival at all sites using the most up to date COVID-19 guidance and case definitions? |  |  |  |  |
| - - 1. Are digital technologies such as telephone triage and video consultations being used to separate patient care pathways for COVID-19 and demands for regular PHC? |  |  |  |  |
| **3.3 Develop new service delivery modalities and innovative platforms and tools** | **Yes** | **No** | **Partly** | **Comments** |
| - - 1. Are PHC services being coordinated with extra-hospital emergency care (e.g., ambulance, telephone helplines to call for urgent care and information and requisition of ambulances for transportation), as well as with social care and public health services for those most vulnerable in society? |  |  |  |  |
| - - 1. Is service delivery at newly established alternative health care sites properly organized for mild or moderately ill patients? |  |  |  |  |
| **3.4 Organize and supervise the care of patients staying at home or in alternate care settings** | **Yes** | **No** | **Partly** | **Comments** |
| - - 1. Are patients and their caregivers being empowered through education on infection prevention and control, isolation and recognition of the need for prompt medical attention? |  |  |  |  |
| - - 1. Is home isolation being prescribed for suspected cases and their contacts? |  |  |  |  |
| - - 1. Is the health situation of people isolated at home or in alternate care sites being monitored, particularly on day 5/6 of the illness (the critical point in the development of the disease)? This may include organizing daily calls/visits by mobile outreach services to check temperatures, blood oxidation levels (where feasible) and other symptoms. |  |  |  |  |
| - - 1. Has a register of people with COVID-19 being treated at home and in alternate care sites been established? |  |  |  |  |
| - - 1. Are COVID-19 patients with highest probability of poor outcomes, irrespective of their legal status, being referred to hospitals? |  |  |  |  |
| - - 1. Using community nurses, community health workers, social services or NGOs, have modalities been established to give special attention to people living alone and to check up on them frequently enough to ensure that their condition does not deteriorate quickly? |  |  |  |  |
| **3.5 Strengthen the interface with the care for people in nursing homes and other closed settings such as refugee camps, prisons and detention centres** | **Yes** | **No** | **Partly** | **Comments** |
| - - 1. Are plans being implemented in nursing homes and other closed settings to ensure early recognition, isolation, care, and source control (prevention of onward spread for an infected person)? |  |  |  |  |
| - - 1. Is access to visitors being restricted and avoided as much as possible? |  |  |  |  |
| - - 1. Are alternatives to in-person visiting being explored (e.g., the use of telephones or video, plastic or glass barriers between residents and visitors)? |  |  |  |  |
| - - 1. Are PHC providers reaching out to nursing homes to incorporate residents into their practice (or dispensarization) lists? |  |  |  |  |
| - - 1. Are staff being trained to recognize the conditions for referral of patients to hospitals? |  |  |  |  |
| - - 1. Are strategies for the rational use of personal protective equipment, including training and monitoring of practices, being implemented? |  |  |  |  |
| **4. Maintain the delivery of essential (non-COVID-19) PHC services during the pandemic** | | | | |
| **4.1 Review and revise the scope of PHC services to be provided during the epidemic to maximize the ability to respond to the COVID-19 epidemic while preserving essential services** | **Yes** | **No** | **Partly** | **Comments** |
| - - 1. Has a list of context-specific essential PHC services that must be maintained been developed?   If so, does this list include only those services that would have a negative impact on the population if postponed/cancelled (e.g., treatment of acute illnesses other than COVID-19, management of patients with NCDs)? |  |  |  |  |
| - - 1. Has a roadmap been established with triggers/thresholds for a phased reduction or reallocation of routine comprehensive service capacity towards essential services? |  |  |  |  |
| - - 1. Have non-essential individual services been postponed or suspended during periods high caseloads of COVID-19? |  |  |  |  |
| - - 1. Have all group activities or services (e.g., education classes, exercise programmes) been either cancelled or moved to an online format? |  |  |  |  |
| - - 1. Has mental health service provision been strengthened, particularly counselling and treatment of depression and anxiety disorders, to address the many sources of stress experienced by all members of society? |  |  |  |  |
| - - 1. Are all PHC providers being advised to be extra alert to signs of domestic violence? |  |  |  |  |
| **4.2 Develop new modalities of work and service delivery to facilitate business continuity of regular PHC** | **Yes** | **No** | **Partly** | **Comments** |
| - - 1. Has effective patient flow (e.g., screening, triage and targeted referral) been established? |  |  |  |  |
| - - 1. Have outreach mechanisms been established as needed to ensure delivery of essential services? |  |  |  |  |
| - - 1. Has tele-working been introduced for staff, particularly for PHC physicians and nurses in self-isolation, to enable them to continue to work? |  |  |  |  |
| - - 1. Has remote/electronic prescribing been introduced? |  |  |  |  |
| - - 1. Have e-consultations or web-based consultations been introduced? |  |  |  |  |
| - - 1. Have e-referrals been introduced? |  |  |  |  |
| - - 1. Has the duration of prescriptions been extended (e.g., to six months or one year) for patients with well-managed chronic conditions, such as hypertension and diabetes? |  |  |  |  |
| **4.3 Develop innovative tools and mechanisms to reduce the burden on PHC providers** | **Yes** | **No** | **Partly** | **Comments** |
| - - 1. Have telephone hotlines dedicated to COVID-19 been established to divert general enquiries away from emergency numbers? |  |  |  |  |
| - - 1. Have basic health assessment online tools or apps been developed? |  |  |  |  |
| - - 1. Have online clearinghouses of information been established to function as repositories for all the relevant guidance and answers to frequently asked questions related to COVID-19? |  |  |  |  |
| - - 1. Have digital tools (e.g., online assessments) been established to reduce the number of cases that need to be assessed by PHC providers?   If so, are these tools available in multiple languages to enable minority groups, migrants and refugees to access them? |  |  |  |  |
| - - 1. Has the administrative burden on PHC providers been reduced and time-consuming reporting requirements eliminated? |  |  |  |  |
| - - 1. Has the regulation requiring first-day sick leave certification by a physician for absences from work been temporarily abolished (e.g. allowingabsences from work for up to the 14 days required for self-isolation after exposure to suspected COVID-19 cases)? |  |  |  |  |
